# Application of Fractional Wave Packet Transform for Robust Watermarking of Mammograms

**DOI:** 10.1155/2015/123790

**Published:** 2015-11-02

**Authors:** Pushpa Mala Siddaraju, Devappa Jayadevappa, Kaliyamoorthy Ezhilarasan

**Affiliations:** ^1^Research Scholar, Jain University and Department of Electronics and Communication Engineering, Sambhram Institute of Technology, Bangalore 560097, Karnataka, India; ^2^Department of Electronic Instrumentation, JSS Academy of Technical Education, Bangalore 560060, Karnataka, India

## Abstract

Exchanging of medical data requires efficient authentication and protection of medical data that can be illegally modified. Watermarking plays an important role in protecting, sharing, and securing medical data. In this work, a robust nonblind medical image watermarking scheme is proposed. The process involves two steps: the embedding and the extraction phase. During the embedding phase, l-level FRWPT is performed on the host image and the watermark is embedded into the modified reference image. In the second phase, inverse FRWPT is performed on the watermarked image to extract the watermark from the watermarked image. The proposed scheme is tested on mammograms images and is subjected to common attacks like Gaussian filtering, median filtering, compression, sharpening, and contrast adjustments. Experimental results show that the proposed scheme is robust.

## 1. Introduction

In the past few decades, there has been a tremendous growth in information and communication technology leading to easier access to any form of digital data including medical data. Modern health care systems produce a large amount of medical data. Medical data are those that are generated from Medical Information System (MIS), Hospital Information System (HIS), Radiology Information System (RIS), and Electronic Patient Records (EPR). Most of the medical diagnosis is based on images from CT scans, X-rays, MRI scans, mammography, and other forms of image modalities. Cancer is the most familiar disease that affects both men and women. The time factor is very important to discover the abnormality issues in target images, especially in various cancer tumors such as the breast cancer. Researchers at the January 1997 Consensus Development Conference presented data discussing the facts related to breast cancers detected by mammography. Their data showed that mammograms have more prognoses than cancers detected by other imaging modalities. Berry [1] has discussed the benefits and risks of mammography. Though the survival rate of a cancer patient is less, it makes a difference for a breast cancer patient. To increase the survival rate of breast cancer patients, mammography is used for earlier detection and treatment stages with reduced risk. These images were effective in detecting breast cancer early.

Doctors' advice and treatment are based on the data the doctor analyses from the mammogram. Nowadays, most of the patients prefer a second/third opinion on the medical analyses received from their doctor. The second/third opinion may be from a doctor located at a different place through telemedicine. There are many such circumstances where telemedicine exists.

Telemedicine applications are those that provide for diagnostics, prescription, consulting, and sometimes conferences to telesurgery. Telemedicine plays an important role in today's world, where the patient's medical data is transmitted over the Internet. The medical data considered may be medical images (mammogram) which has to be shared or transmitted over Internet. This medical data is crucial data and is at the risk of unauthorized access or manipulation. Authenticating as well as protecting it is essential since critical judgment is done on medical images especially mammograms during breast cancer detection.

Cryptography, steganography, and watermarking are such schemes that are used to protect digital data. Cryptography and steganography are less robust or partially robust to digital data modifications. This scenario is compensated in medical imaging through watermarking. Digital image watermarking is a technique of embedding a watermark (say logo) into the host image for dealing with security issues. Digital image watermarking can be extended to medical images too. Medical image watermarking deals with medical data authentication, ownership, security, source identification, and patient identification. When dealing with EPR, it is mandatory to consider confidentiality to access the information, availability of the data to be used when required, and reliability of the data. These are maintained through security issues, namely, integrity, availability, authentication, and confidentiality nonrepudiation [2]. Medical image watermarking provides for security at the origin reducing piracy of crucial medical data and also protects medical documents or images that can be illegally modified. The medical image watermarking scheme designed requires being robust, secure, and imperceptible. Part of the medical information, the Region of Interest (ROI), is crucial and must not be altered. This region provides the information for the doctor to diagnose and treat the patient. Hence this region has to be secured.

Watermarking schemes can be broadly classified as time domain and frequency domain schemes. Several watermarking schemes exist, and these schemes adopt both additive and multiplicative approaches in time domain. These schemes adopted SS (Spread Spectrum) [3], DCT (Discrete Cosine Transform) [4], DFT (Discrete Fourier Transform), DWT (Discrete Wavelet Transform) [5], SVD (Singular Value Decomposition) [6], Ridgelets [7], and contourlets [8] in frequency domain. Lim et al. [9] proposed a watermarking scheme to verify the integrity and authenticity of CT scan images. Here, the watermark was processed as an input to the hash function. Raúl et al. [10] proposed a pixel based watermarking embedding scheme using spiral scan.

Coatrieux et al. [11] have identified three different schemes to watermark a medical image:

- Embedding the information within the Non-Region of Interest (NROI): this method [12] generally places the watermark in the gray portions of the image. These methods were perceptible. During application of salt and pepper noise, this method may seem too annoying to the physician.
- These schemes deal with reversible watermarking and were better suited for integrity control and data hiding [13].
- These methods normally minimize distortion. This was achieved by replacing some of the image details.

Piva et al. [14] have proposed a method where the physician selects the maximum power of the watermark under the level of interference with diagnosis. This method was robust. Wakatani [15] proposed a scheme wherein the watermark was embedded in the NROI (Non-Region of Interest) by adopting DWT. The nonwatermarked area was easily prone to attacks. Giakoumaki et al. [16] proposed a robust multiple medical image watermarking scheme using 4-level DWT. The watermark was embedded at different decomposition level and was tested on ultrasound images. Another multiple medical image watermarking was proposed by Memon et al. [17]. The watermark was embedded by separating the ROI and NROI. This scheme was both robust and fragile. Watermark embedded in the NROI is visible. Hence, another option is to embed the watermark in the ROI. This further preserves the ROI from unwanted manipulations. Medical image watermarking schemes must consider computational complexities which may lead to time delays for the physician and are still in the early stages of development. It is difficult to evaluate watermark interferences with diagnosis.

In this paper, a robust medical image watermarking scheme using fractional wavelets is proposed. The proposed scheme is divided into two stages, namely, the embedding stage and the extraction stage. FRFT (Fractional Fourier Transform) has good reconstruction capability compared to FFT (Fast Fourier Transform). When FRFT is combined with WPT (Wave Packet Transform), it has the capability to retain the coefficients after attacks. Three levels of security are provided. Firstly, Arnold Transform is applied to the host image and the host image is scrambled. Secondly, the value of the transform order *β* is used as the key. According to this key the reference image is generated. This provides for the security of the watermark. The transform order *β* is user defined and hence randomly chosen. Thirdly, the position of all frequency subbands is changed at each level using some secret rule known only to the user or creator. The adversary cannot extract watermark without accessing the reference image. Quality of the extracted watermark to the original watermark is directly proportional to degradation of the image quality.

A brief introduction on medical image watermarking is introduced in Section 1. This section also deals with some literature survey on the different schemes available. The paper is organized as follows. Sections 2 and 3 describe Fractional Wave Packet Transform and Arnold Transform, respectively.  Section 4 describes the proposed method.  Section 5 presents the results and discussions of the proposed method. In Section 6 we conclude the work presenting the future enhancements to obtain more robust medical image watermarking schemes.

## 2. Fractional Wave Packet Transform

Based on the idea of Fractional Fourier Transform (FRFT) and Wave Packet Transform (WPT), Fractional Wave Packet Transform (FRWPT) was introduced by Huang and Suter [18]. Mathematically, for an input signal *x*(*t*) represented along the time axis and its Fourier Transform represented along the frequency axis, FRFT, *F*_*α*_(*t*, *u*), of the input signal, *x*(*t*), is given by

(1) $$\begin{array}{c} F_{\alpha }\left(\;t,u\;\right)=\overset{-\infty }{\underset{+\infty }{\int }}C_{\alpha }\left(\;t,u\;\right)x\left(\;t\;\right)dt, \end{array}$$

where *C*_*α*_(*t*, *u*) is the Fourier transformation kernel. FRFT corresponds to a rotation by an angle.

The Wave Packet Transform (WPT) is a combination of Short Time Fourier Transform (STFT) and Continuous Wavelet Transform (CWT). WPT (*W*_*α*_) of an input signal, *x*(*t*), is given by

(2) $$\begin{array}{c} W_{\alpha }=\frac{1}{\sqrt{2\pi \alpha }}\overset{+\infty }{\underset{-\infty }{\int }}\exp\left(\;-jut\;\right)\psi \left(\;\frac{t-\beta }{\alpha }\;\right)x\left(\;t\;\right)dt. \end{array}$$

WPT can also be defined as the FT of a signal windowed by a wavelet, dilated by *α* and translated by *β*.

FRWPT, *W*_*α*_(*u*, *a*, *b*), for a given input signal *x*(*t*) can be defined as

(3) $$\begin{array}{c} W_{\alpha }\left(\;u,a,b\;\right)=\frac{1}{\sqrt{a}}\overset{-\infty }{\underset{+\infty }{\int }}C_{\alpha }\psi \left(\;\frac{t-b}{a}\;\right)x\left(\;t\;\right)dt. \end{array}$$

The FRWPT is a function of time, frequency, and scale.

The computation of FRWPT corresponds to the following steps as explained by Huang and Suter [18]:

1. A product by a wavelet.
2. A product by a chirp.
3. A Fourier Transform.
4. Another product by a chirp.
5. A product by a complex amplitude factor.

**Figure 1:**
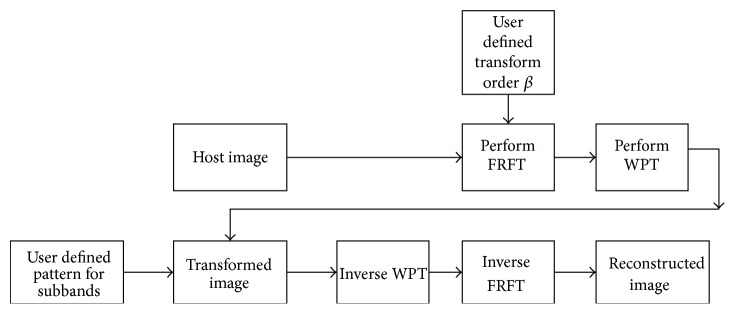
Simplified computation efficient FRWPT.

For computational purposes, a simple implementation of FRWPT is used as shown in Figure 1.

The computational algorithm can be described as follows:

(1) The transform order of *β* is used for the FRWPT. This is obtained by trial and error method. If FRWPT is applied to the original signal, and the transformed signal is reconstructed from inverse FRWPT, then the transform order *β* is that value which results in minimum mean square error between the original and the reconstructed signal. The process may sometimes be long and cumbersome due to its trial and error approach, but it is limited only to once per signal.
(2) FRFT is performed on the input signal, with user defined transform order *β*, followed by Wavelet Packet Transform. This phase is the decomposition phase.
(3) Inverse Wavelet Packet Transform and inverse FRFT are performed on the transformed signal with transform order *β*. This phase is the reconstruction phase.

## 3. Arnold Transform

To further improve the security and improve the proposed watermarking scheme, the host image is preprocessed (scrambled) by applying Arnold Transform. l-level FRWPT is applied to the Arnold Transformed image. One of the properties of the Arnold Transform is periodicity. Due to this property, the image can be easily recovered after *n*-permutations.

Consider an image of size *N* × *N*; the Arnold Transform is defined by

(4) $$\begin{array}{c} \left[\begin{array}{c} x^{\prime }\\ y^{\prime } \end{array}\right]=\left[\begin{array}{cc} 1 & 1\\ 1 & 2 \end{array}\right]\left[\begin{array}{cc} 1 & 1\\ 1 & 2 \end{array}\right]\left[\begin{array}{c} x\\ y \end{array}\right]\mathrm{mod}\left(\;N\;\right), \end{array}$$

where *x*, *y* are the coordinates of the image and *x*′, *y*′ are the coordinates after scrambling.

## 4. Proposed Work

**Figure 2:**
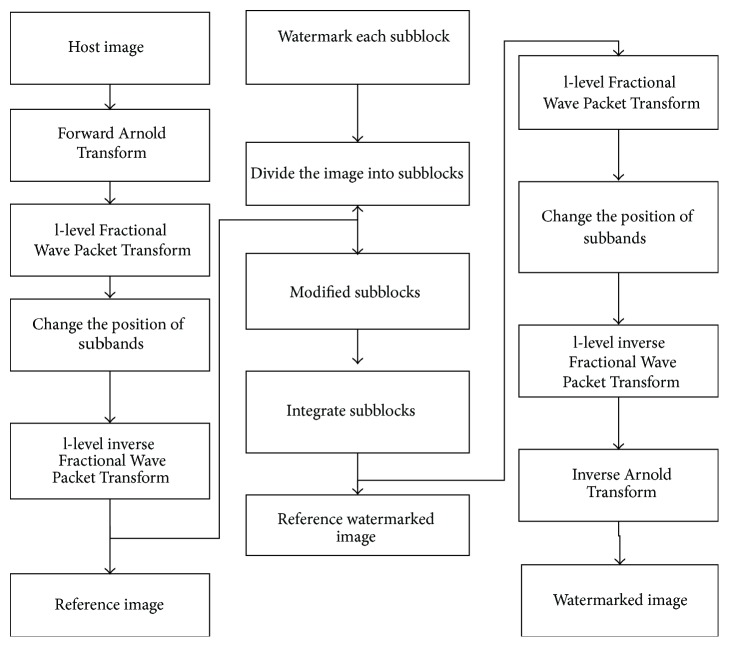
Watermark embedding.

**Figure 3:**
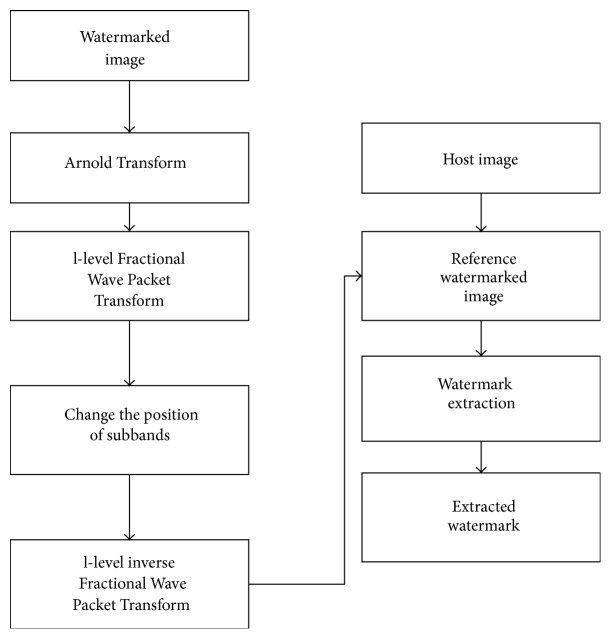
Watermark extraction process.

The proposed scheme can be divided into two phases: the embedding phase and the extraction phase. In the embedding phase, the host image is converted to a reference image and the watermark is embedded on the reference image. During the extraction phase, the watermark is first extracted and then the host image is obtained from the reference image. The watermark used is a grey scale logo/image. The embedded watermark is smaller than the host image by a factor raised to the power of 2 along both the directions. The proposed embedding and extraction scheme is shown in Figures 2 and 3, respectively. Assuming that *I*(*x*, *y*) represents the original image of size *P* × *Q*, *W*(*x*, *y*) represents the watermark of size *p* × *q*; the embedding and the extraction phase can be detailed as follows.

### 4.1. Watermark Embedding

The proposed watermarking embedding process can be outlined as follows:

(1) Scramble the host image by applying Arnold Transform.
(2) Apply l-level Fractional Wave Packet Transform to the Arnold Transformed image. The transform order *β* is chosen, defined by the creator. The subbands obtained are HH, HL, LH, and LL.
(3) Change the position of the subbands. The pattern to swap/change the image subband is defined by the creator.
(4) Apply l-level inverse fractional wavelet transform to obtain the reference image *I*_ref_(*x*, *y*) and then divide the reference image into subblocks.
(5) Apply watermark to the subblocks to obtain the modified reference watermarked image.
 The modified subblocks are represented by
  (5) $$\begin{array}{c} W_{\text{ref}}=I_{\text{ref}}\left(\;x,y\;\right)+\beta W\left(\;p,q\;\right), \end{array}$$
 where *α* denotes the strength of the watermark. Integrate the subblocks to obtain the reference watermarked image, *W*_ref_^*∗*^(*x*, *y*).
(6) Perform l-level fractional wavelet transform.
(7) Change the position of subbands. Perform l-level inverse fractional wavelet transform.
(8) Apply Arnold Transform to scramble back the image to obtain the watermarked image, *W* ^*∗*^(*x*, *y*).

### 4.2. Watermark Extraction

The watermark extraction process can be outlined as follows:

(1) Apply Arnold Transform on the watermarked image *W* ^*∗*^(*x*, *y*).
(2) Perform l-level fractional wavelet transform on the Arnold Transformed image.
(3) Change the positions of all the subbands.
(4) Perform l-level inverse fractional wavelet transform to obtain the watermarked reference image, *W*_ref_^*∗*^(*x*, *y*).
(5) Extract the watermark, *W*(*x*, *y*).
(6) Perform l-level fractional wavelet transform on the watermarked image, *W* ^*∗*^(*x*, *y*).
(7) Change the positions of all the subbands.
(8) Perform l-level inverse fractional wavelet transform to obtain the host image, *I*(*x*, *y*).

## 5. Results and Discussions

**Figure 4:**
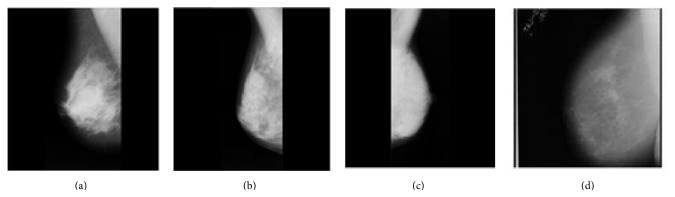
Host image: (a) mdb001, (b) mdb017, (c) mdb054, and (d) mdb153.

**Figure 5:**
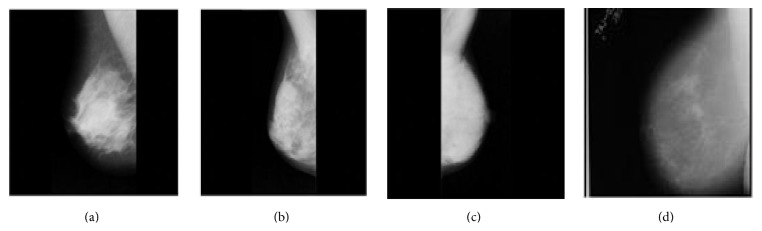
Watermarked image: (a) mdb001, (b) mdb017, (c) mdb054, and (d) mdb153.

The performance of the proposed scheme is explored and analysed using Matlab. A number of experiments are performed on mammogram images from the MIAS database. The images chosen are mdb001, mdb017, mdb054, and mdb153.  Figure 4 shows the host images used. The watermark used is the BMW logo (any grey scale image can be used); reference image is obtained using 2-level decomposition of FRWPT. Since 2-level decomposition gives a block size of 64 × 64, the watermark is embedded 16 times into the modified reference image to get the watermarked reference image.  Figure 5 shows the watermarked image. The watermarked image quality is measured using the evaluation metrics PSNR and SSIM.  Table 1 shows the values of PSNR and SSIM values of the watermarked image with reference to the original image without any attack. The watermarked image is subjected to common attacks to investigate the robustness of the proposed scheme. The attacks considered are average and median filtering, Gaussian noise, salt and pepper noise, cropping, resizing, rotation, and sharpening attacks. The extracted watermark is compared with the original watermark. The performance of the watermarking algorithm under these attacks is tabulated in Tables 2 and 3.

The watermarked image is subjected to 13 × 13 median filtering and Weiner filtering. Median filtering attack degrades the image quality. The extracted watermark quality is also degraded. When the image is subjected to Weiner filtering the image quality is not degraded and the watermark quality is recognizable. This is due to addition of noise which degrades the quality of the image and hence the watermark extracted is degraded. Similar effects are also seen when Gaussian noise and salt and pepper noise are added. Hence, the extracted watermarks under noisy environments are still recognizable.

Storage of medical image requires the images to be compressed. The proposed algorithm is observed to be robust under compression attack. Cropping is done by deleting certain number of rows and columns. Since a large part of the image was cropped (200 × 200 pixels towards upper left corner of the image), most of the medical information was removed. But this can be further modified to crop only the Non-Region of Interest, preserving important medical data.

**Figure 6:**
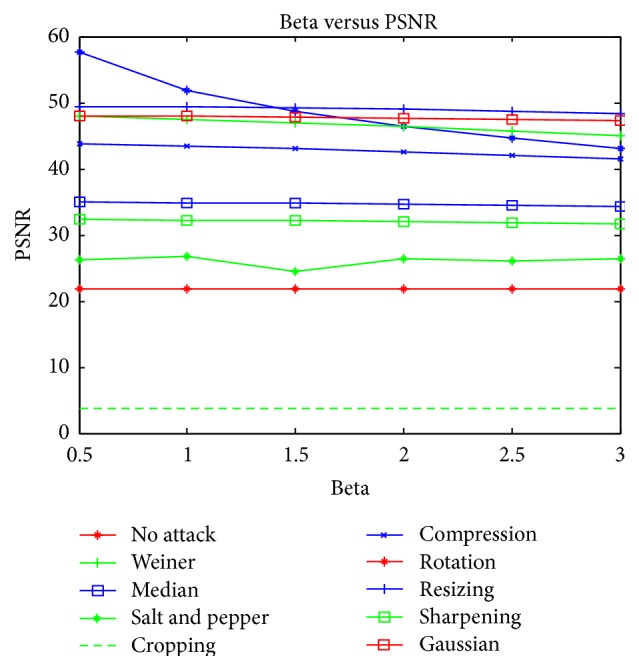
Relation between *β* and PSNR for various attacks.

**Figure 7:**
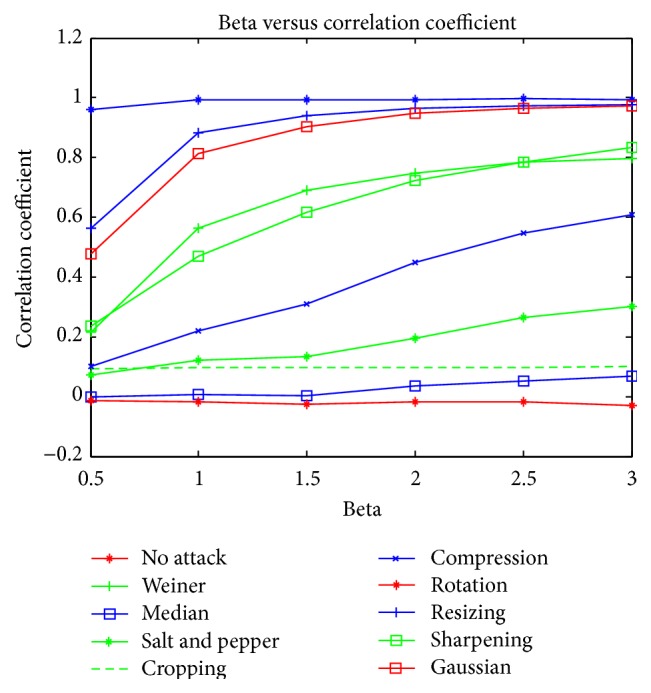
Relation between *β* and correlation coefficient for various attacks.

The image was rotated by an angle of 5 degrees towards the left and right. The results for 5-degree left rotation are tabulated. The proposed scheme could also withstand rotation effect. The proposed algorithm was also tested for sharpening and contrast adjustments and results show that the proposed scheme could withstand these results. Graphical analysis of the proposed scheme under various common attacks discussed above is depicted in Figures 6 and 7. The graph shows the relation between PSNR and correlation coefficient for different values of *β*, where *β* denotes the strength of the watermark. It is observed that the proposed watermarking scheme is not image variant and maintains a linear relationship for most of the mammogram images used.

## 6. Conclusion

In this work, a robust nonblind medical image watermarking scheme for mammograms was proposed applying FRWPT. Experimental results show that the scheme is robust to common attacks like the compression attacks, interference attacks, and signal processing attacks. The experimental results are tabulated adopting watermark evaluation metrics PSNR and SSIM. The scheme can be further improved by embedding the watermarking in the NROI. But embedding the watermark in the NROI will make the watermark visible. It is vital to adopt imperceptible watermarks to ensure security issues of integrity and authenticity.

## Figures and Tables

**Table 1:** Evaluation metrics of proposed algorithm.

Image	mdb001	mdb017	mdb054	mdb153
PSNR	51.9226	52.1666	52.1268	50.5253
SSIM	0.9876	0.9868	0.9863	0.9930

**Table 2:** Correlation coefficients of extracted watermarks (*β* = 1).

Attack	mdb001 PSNR	Correlation coefficient	mdb017 PSNR	Correlation coefficient	mdb054 PSNR	Correlation coefficient	mdb153 PSNR	Correlation coefficient
No attack	51.9226	0.99167	52.1666	0.9911	52.1268	0.9863	50.5253	0.9990
Weiner	47.4623	0.56504	47.8532	0.5728	50.1466	0.6754	44.2345	0.5348
Median filtering	34.8623	0.00565	35.5175	0.0301	36.9109	0.03534	22.6301	0.0155
Salt and pepper noise	26.7163	0.12153	46.1605	0.7378	26.4035	0.1157	27.3673	0.1713
Cropping	3.84305	0.09536	3.4014	0.0762	3.3462	0.0764	3.6300	0.100
Compression	43.4441	0.21957	44.0487	0.1998	46.1554	0.2317	41.5755	0.2856
Rotation	21.8898	−0.0158	20.0117	−0.0046	19.6339	−0.0011	17.9435	0.0269
Resizing	49.4074	0.88312	47.210	0.8365	46.8850	0.8116	43.4394	0.8355
Sharpening	32.2288	0.46943	32.0762	0.4280	33.1590	0.4798	27.4106	0.4340
Gaussian filter	47.9443	0.81130	46.1605	0.7378	45.3153	0.7336	42.0658	0.7408

**Table 3:** Correlation coefficients of extracted watermarks (mdb001).

*β*	*β* = 0.5		*β* = 1.5		*β* = 2.0		*β* = 2.5	
Attack	PSNR	Correlation coefficient	PSNR	Correlation coefficient	PSNR	Correlation coefficient	PSNR	Correlation coefficient
No attack	57.6742	0.9586	48.7639	0.9951	46.4094	0.9946	44.5710	0.9970
Weiner	47.9247	0.2171	46.9645	0.6886	46.3327	0.7492	45.7129	0.7842
Median filtering	34.9318	0.0002	34.7781	0.0037	34.6527	0.0366	34.5133	0.05360
Salt and pepper noise	26.2601	0.0704	24.4059	0.1352	26.3242	0.1933	25.9943	0.2644
Cropping	3.8383	0.0932	3.8466	0.0956	3.8505	0.0972	3.8541	0.0981
Compression	43.7967	0.1001	43.0275	0.3107	42.5067	0.4475	42.0062	0.5454
Rotation	21.8911	−0.0137	21.8871	−0.0264	21.8810	−0.0165	21.8738	−0.0162
Resizing	49.458	0.5626	49.2708	0.9406	48.9864	0.9642	48.7016	0.9735
Sharpening	32.3031	0.2374	32.1365	0.6153	32.0112	0.7222	31.8713	0.7826
Gaussian filter	48.0211	0.4770	47.8309	0.9038	47.6739	0.9463	47.4890	0.9632
